# Consider the unexpected! An overlooked, elusive, rare but dramatic diagnosis: anorectal melanoma

**DOI:** 10.1007/s13304-025-02367-y

**Published:** 2025-08-28

**Authors:** Rossella Melcarne, Chiara Eberspacher, Massimiliano Mistrangelo, Pietro Quaglino, Rebecca Senetta, Arcangelo Picciariello, Leonardo Vincenti, Daniela Rega, Paolo Delrio, Corrado Caracò, Mariarosaria Portinaio, Stefano Arcieri, Giovanni Paolino, Santo Raffaele Mercuri, Carmen Cantisani, Chiara Scorziello, Tal Deborah Engel, Laura Giacomelli, Marco Biffoni, Domenico Mascagni

**Affiliations:** 1 Department of Translational and Precision Medicine, Sapienza University of Rome, Viale del Policlinico, 155, 00161 Rome, Italy; 2 Department of General Surgery, Sapienza University of Rome, Viale del Policlinico, 155, 00161 Rome, Italy; 3 Surgical Science Department, Centre of Minimal Invasive Surgery, Città Della Salute E Della Scienza Hospital, University of Turin, Turin, Italy; 4 Department of Medical Sciences, Sections of Dermatology, University of Turin, Turin, Italy; 5 Pathology Unit, Department of Medical Sciences, University of Turin, Turin, Italy; 6 Department of Experimental Medicine, University of Salento, Lecce, Italy; 7 Surgical Unit, IRCCS de Bellis, Castellana Grotte, 70013 Bari, Italy; 8 Colorectal Surgical Oncology, Department of Abdominal Oncology, Istituto Nazionale Tumori-IRCCS “Fondazione G. Pascale”, Naples, Italy; 9 Division of Surgery of Melanoma and Skin Cancer, Istituto Nazionale Tumori ‘Fondazione Pascale’ IRCCS, Naples, Italy; 10 Department of Clinical Medicine and Surgery, University of Naples Federico II, Via S. Pansini, 5, 80131 Naples, Italy; 11 Unit of Dermatology, IRCCS Ospedale San Raffaele, Milan, Italy; 12 Faculty of Medicine and Surgery, Vita-Salute San Raffaele University, Milan, Italy; 13 Department of Dermatology, Policlinico Umberto I, Sapienza University of Rome, Rome, Italy; 14 Sant’Andrea Hospital, Sapienza University of Rome, Rome, Italy; 15 Department of General and Specialized Surgery and Anesthesiology , Sapienza University of Rome, Viale del Policlinico, 155, 00161 Rome, Italy

**Keywords:** Anal melanoma, Anal cancer, Mucosal melanoma, Wide local excision, Multidisciplinary management, Rare malignancy

## Abstract

Background: Anorectal melanoma (AM) is a rare and aggressive malignancy, often misdiagnosed due to its clinical resemblance to benign anorectal conditions. Early diagnosis remains challenging, with a poor prognosis and high rates of metastasis at presentation. Methods: We conducted a retrospective multicenter study of 21 patients diagnosed with AM between 2013 and 2023 across four high-volume Italian surgical centers. Patients were stratified into two groups based on whether AM was suspected at initial evaluation (Group A) or incidentally diagnosed after surgery for presumed benign disease (Group B). Clinical, diagnostic, treatment, and outcome data were analyzed. Results: Only 24% of patients had AM suspected at first presentation. These patients were younger (median age 49 vs. 70 years) and had larger, more readily identifiable tumors. However, nodal and distant metastases were equally frequent in both groups (lymph node metastases: 52.4%; distant metastases: 19%). Most patients underwent wide local excision (71.4%), while only one required abdominoperineal resection. Postoperative recurrence occurred in 47.6% of cases. Median survival was 11 months in Group A and 24 months in Group B. In 90.5% of cases, previous specialist consultations had failed to achieve timely diagnosis, highlighting missed diagnostic opportunities. Conclusions: AM is frequently overlooked due to its rarity and non-specific presentation. Earlier recognition alone may not improve outcomes, but systematic histopathological assessment, targeted biopsy, and multidisciplinary management remain essential. Conservative surgery with early use of systemic therapy should be prioritized when feasible.

## Introduction

Anorectal melanoma (AM) is a rare and aggressive malignancy, often misdiagnosed due to its non-specific symptoms and clinical resemblance to benign anorectal conditions [1]. As a result, it is frequently detected only after surgery for presumed benign disease, such as hemorrhoidectomy [2–4]. Amelanotic variants, accounting for up to 10%–30% of cases, further complicate recognition and contribute to incidental diagnoses [5–7]. This malignancy requires a high degree of clinical suspicion, particularly in patients presenting with rectal bleeding or perianal masses [8–10]. Missed or delayed diagnoses result in lost opportunities for timely treatment, as highlighted by the variability in its presentation [3, 8, 11]. To reduce the risk of missed cases, a routine histopathological examination of all anorectal surgical specimens, even those appearing benign, is strongly recommended [12]. Biopsy, preferably multiple, remains indispensable, especially in the absence of pigmentation [13–15]. The differential diagnosis should include rectal adenocarcinoma, leiomyosarcoma, epidermoid carcinoma, carcinoid tumors, hypertrophic papillae, and other polypoid lesions, most notably thrombosed hemorrhoids, which may clinically and morphologically mimic malignant lesions [3, 16]. A high index of suspicion and the correct use of diagnostic tools are critical for improving early detection and outcomes [8–10]. Most patients already at the time of the first diagnosis present locally advanced disease or distant metastases, and ether recurrence after initial response to treatment is common [8]. These factors underline both the diagnostic complexity, and the biologically aggressive nature of AM. Surgical treatment is often extensive, though its actual benefit remains debated [17].

Epidemiologically, AM accounts for approximately 1.5% (range: 0.3–3%) of all melanomas and 1–3% of anorectal malignancies other than adenocarcinomas [1–3]. Its incidence is markedly lower than that of squamous carcinoma and rectal adenocarcinoma, with reported ratios of 1:8 and 1:250, respectively [18, 19]. It most commonly affects patients in the sixth and seventh decades of life [1, 20, 21], with conflicting data regarding sex distribution: while some studies report no significant difference, others suggest a slight female predominance [22]. Mucosal melanoma is reported less frequently in individuals with darker pigmentation, possibly due to the antioxidant rather than photoprotective effects of melanin [23, 24]. Histologically, AM arises from melanocytes located in the squamous and transitional zones of the anal canal, particularly at the dentate line, with possible proximal extension into the rectum [9].

Heightened clinical suspicion is essential to avoid diagnostic delays, and AM should be considered in the differential diagnosis of any atypical anorectal lesion [3, 8, 11]. Once identified, management should be entrusted to high-volume referral centers with established multidisciplinary pathways, given the complexity of treatment and the poor prognosis associated with this disease [25].

**Fig. 1:**
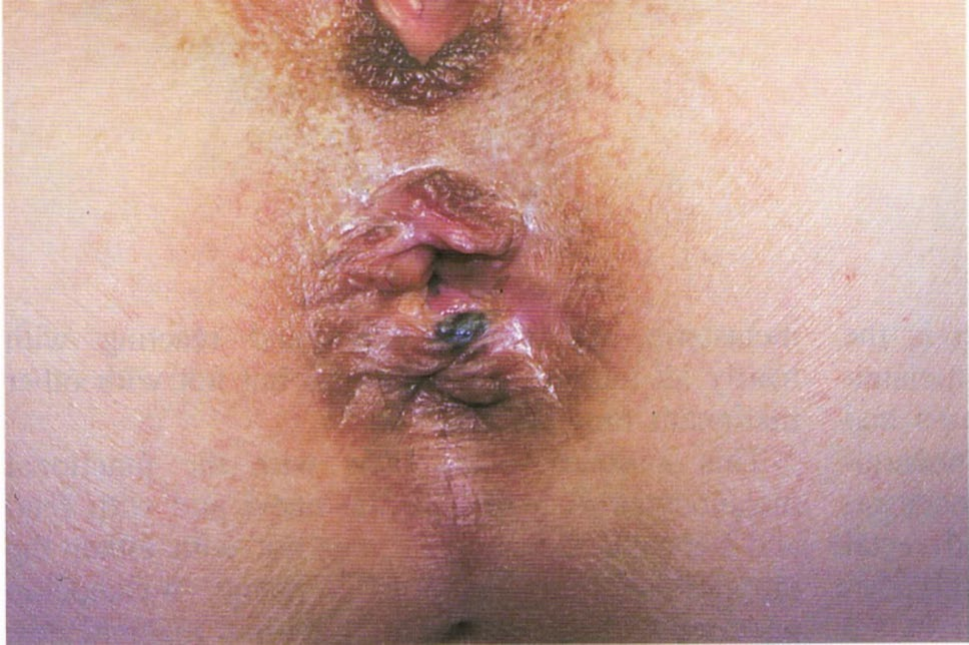
Anorectal melanoma. Aspect of the lesion at clinical examination. The lesion shows irregular borders and pigmentation, indicative of malignancy. This image was previously published in:-Anorectal melanoma: Clinico-pathological features of a rare and aggressive malignancy in a multicentric study. J Eur Acad Dermatol Venereol. 2025 Mar 18. https://doi.org/10.1111/jdv.20647. PMID: 40,103,352.-Anal Canal Cancer (Messinetti S., Giacomelli L., Manno A.), 1993. Reproduced with permission from the authors

**Fig. 2:**
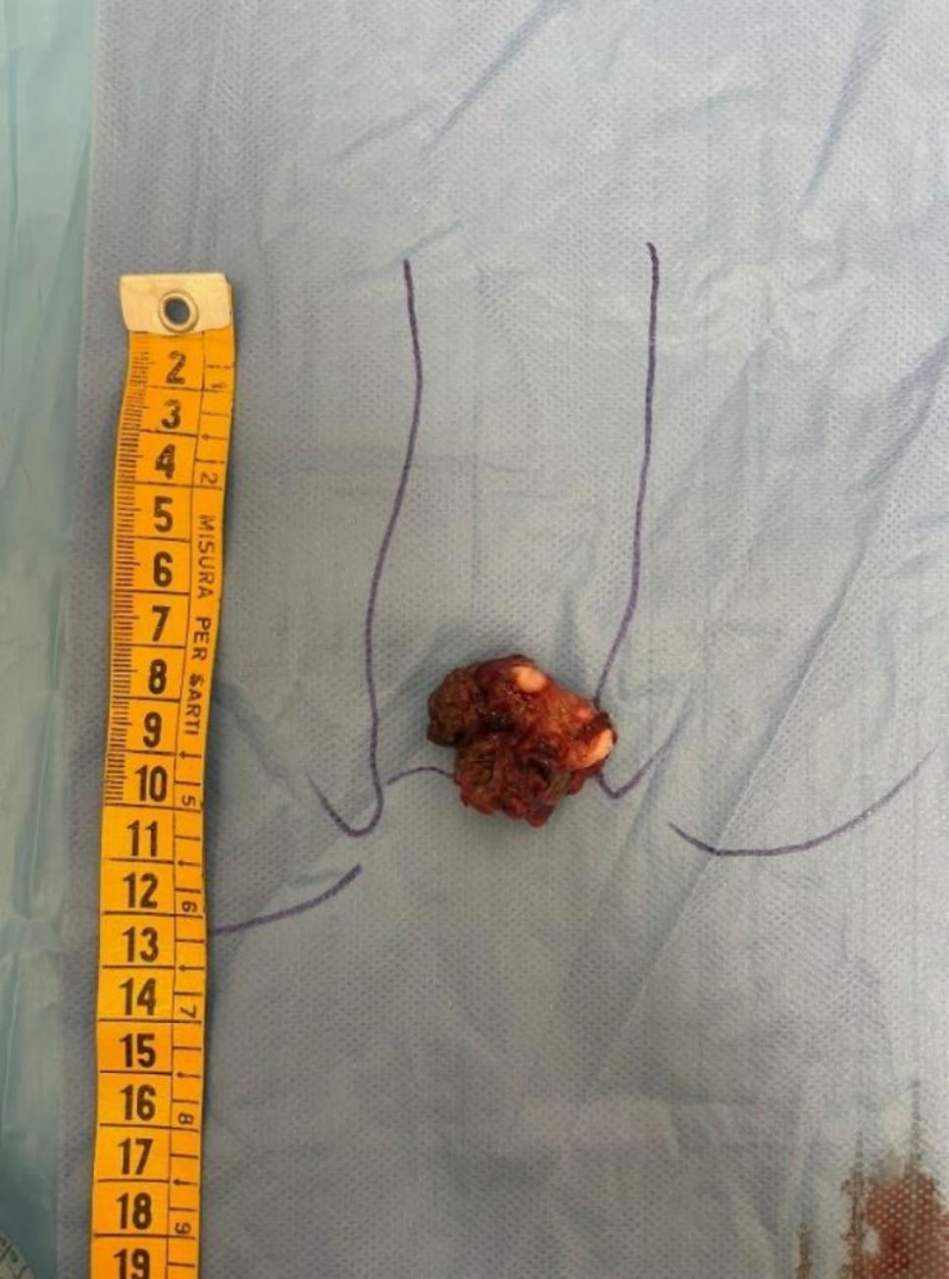
Surgical specimen of anorectal melanoma (**A**). Macroscopic biopsy of an anorectal melanoma lesion (**B**). The specimen highlights the heterogeneity of the tumor, with areas of pigmentation and irregular tissue architecture

**Fig. 3:**
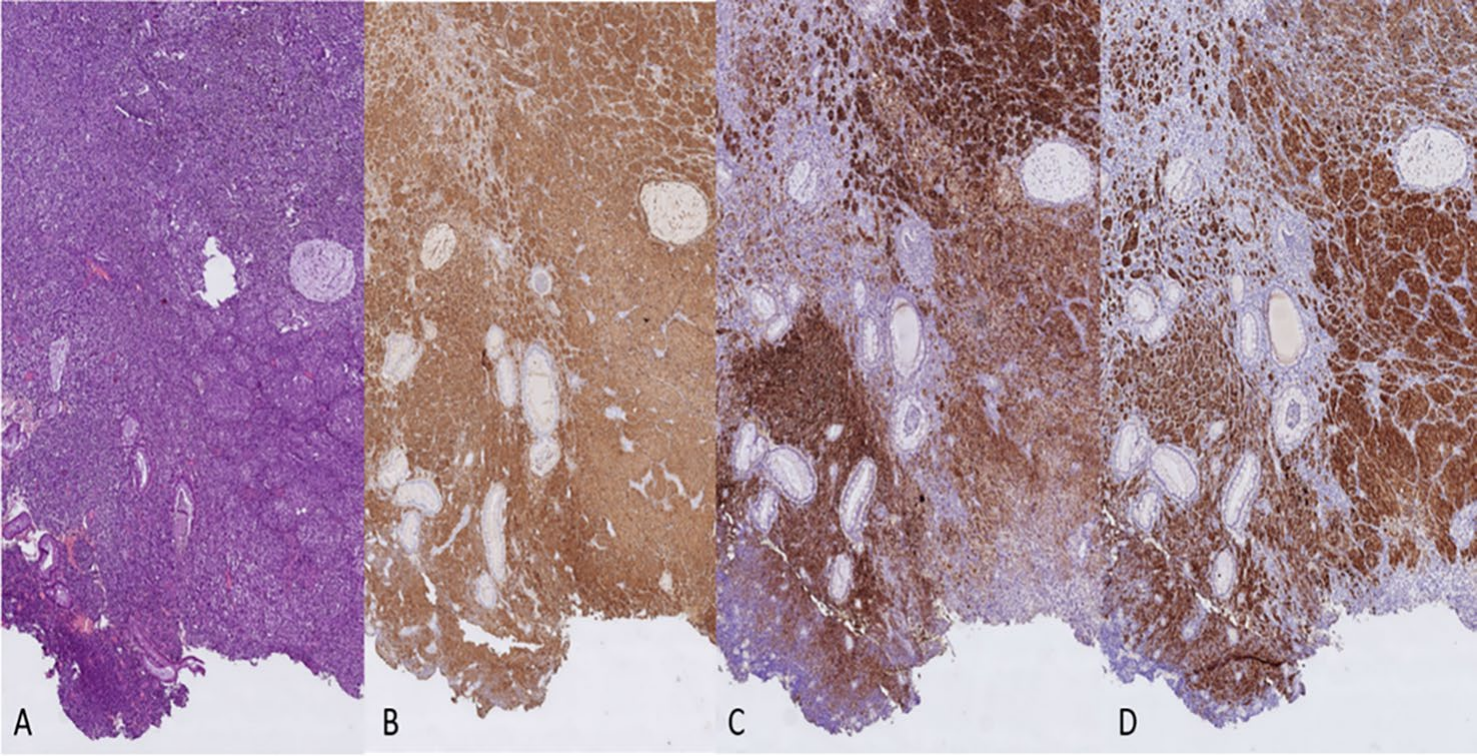
Histological slides of an anorectal melanoma. Hematoxylin–eosin stained section of anal glands and malignant cells arranged in solid nests with pleomorphic nuclei, prominent nucleoli and occasional melanin pigment in the cytoplasm (**A**); Immunohistochemical analysis of the anal melanoma showing positivity for S100 (**B**), HMB45 (**C**) and MelanA (**D**) immunohistochemistry. This image was previously published in: Anorectal melanoma: Clinico-pathological features of a rare and aggressive malignancy in a multicentric study. J Eur Acad Dermatol Venereol. 2025 Mar 18. https://doi.org/10.1111/jdv.20647. PMID: 40103352.

**Fig. 4:**
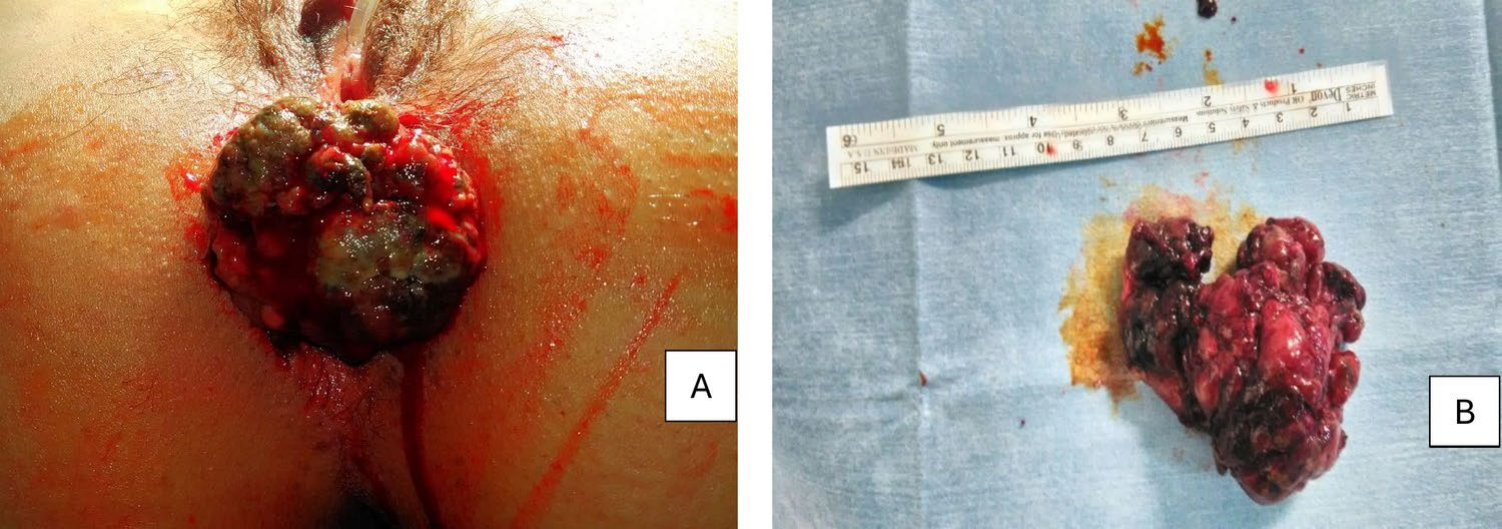
Clinical presentation of anorectal melanoma manages in this surgical series. Bleeding mass (**A**). Macroscopic aspect of the lesion (**B**)

**Fig. 5:**
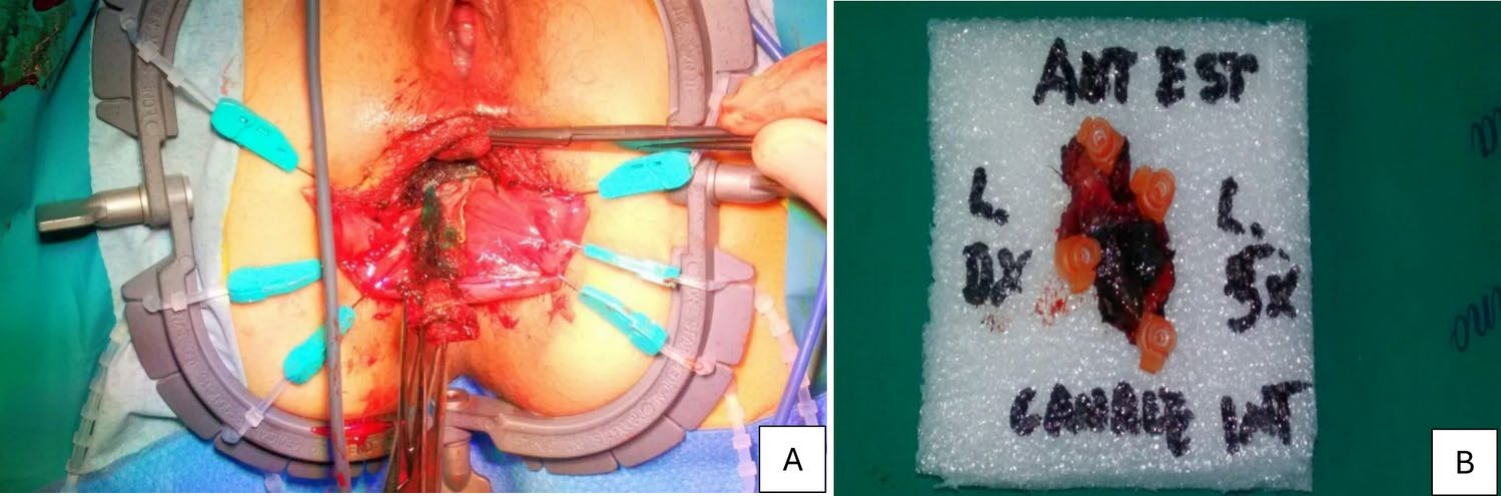
Clinical presentation of anorectal melanoma manages in this surgical series. Intraoperative approach. Pigmented Lesion (**A**). Macroscopic aspect of the lesion (**B**)

This study does not merely aim to report clinical outcomes, but to shed light on the diagnostic journey of patients with AM, highlighting how clinical vigilance and systematic pathology protocols may impact early detection, even when prognosis remains poor. While the study design is conventional, its strength lies in addressing a rarely discussed and diagnostically elusive malignancy. As such, it represents a frontier contribution that may foster greater clinical awareness and stimulate further research on AM (Fig 1, 2, 3, 4, 5)

## Materials and methods

This retrospective multicenter study included cases of AM treated between January 2013 and December 2023 at four high-volume proctological and oncological surgery centers in Italy: AOU Policlinico Umberto I of Rome, Sapienza University of Rome; Città della Salute e della Scienza Hospital, University of Turin; IRCCS de Bellis, Castellana Grotte, Bari; and the Istituto Nazionale Tumori - IRCCS “Fondazione G. Pascale,” Naples. These centers were selected for their referral role in managing rare and complex anorectal malignancies, providing a geographically and clinically diverse cohort.

All patients diagnosed with primary anorectal melanoma at the participating centers over the past ten years were considered for inclusion. To be eligible, patients had to have a histopathological diagnosis of anorectal melanoma and available records regarding initial treatment and follow-up of at least 12 months or until death. Patients were excluded if they denied consent for data processing, had incomplete clinical documentation, or were lost to follow-up immediately after diagnosis.

### Data collection

Demographic, clinical, and pathological data were retrieved from institutional records. For each patient, the following variables were collected:

- Age and sex;
- Presenting symptoms;
- Tumor characteristics (location, size, histological subtype);
- Diagnostic procedures (imaging)
- Treatment modalities (surgical approach, adjuvant therapies);
- Follow-up outcomes (recurrence, metastases, survival).

All data were anonymized and securely stored in accordance with ethical and privacy regulations.

### Statistical analysis

Descriptive statistics were performed using MedCalc software. Continuous variables are reported as medians and ranges; categorical variables are expressed as absolute frequencies and percentages. Given the small sample size and high risk of model overfitting, multivariate analysis was not performed. Results from univariate comparisons should be interpreted with caution and are meant to be exploratory.

## Results

This study included twenty-one patients diagnosed with AM over a 10-year period (2012–2022). The median age at diagnosis was 66 years old (range: 37–87), with a predominance of female patients (15/21 patients; 71.4%). (Table 1).

**Table 1:** Baseline and clinical features at the diagnosis in Patients with Early (Group A) vs. Incidental Diagnosis of Anorectal Melanoma (Group B)

	Group A—Diagnosis suspected at first evaluation (n = 5)	Group B—Diagnosis made after surgery (n = 16)
Median age (range) yrs	49 (37–72)	70 (46–87)
Sex (F/M)	3:2	12: 4
Initial symptom (Nr.)		
Rectal bleeding	3	8
Mass	2	6
Anal pruritus	–	2
Weight Loss	–	1
Amelanotic	0	2
Location (Nr.)		
Perianal	1	2
Anal canal	4	13
Rectum	-	1
Initial Tumor size, Median (mm)	20	15
Previous proctologic and/or dermatologic assessments	4	15
Lymph node metastases at diagnosis (Nr.)	3	6
Inguinal	1	6
Pelvic	1	2
Abdominal/Thoracic	1	0
Distant metastases at diagnosis	2	2
Systemic spread		
Liver	1	2
Lung	1*	0
Brain	1*	0
Pre-operative Imaging (Nr)		
US	5	5
CT	5	9
MRI	3	7
PET	5	2

Comparison of clinical and prognostic features in patients with anorectal melanoma, stratified by diagnostic timing. *yrs* years, *F* female, *M* male, *N/Nr* number, *US* ultrasound, *CT* computed tomography, *MRI* magnetic resonance imaging, *PET* positron emission tomography

* Indicates data referring to the same patient

To better characterize the diagnostic pathways and their potential impact on clinical outcomes, patients were stratified into two groups based on whether anorectal melanoma was suspected at initial evaluation (Group A) or incidentally diagnosed following surgery for presumed benign conditions (Group B). This classification aimed to explore differences in presentation, management, and prognosis associated with the timing of diagnosis.

The most common presenting symptoms were rectal bleeding (11/21 patients; 52.4%) and the presence of a palpable mass (8/21 patients; 38%), while pruritus and unintentional weight loss were each reported in one patient (1/21 patients; 4.8%). In only five patients (5/21 patients; 24%) was AM clinically suspected at first presentation. These patients were markedly younger (median age Group A: 49 years) compared to those whose diagnosis was incidental (median age Group B: 70 years). Tumors in the early-diagnosis group were more frequently located in the anal canal or anorectal region. Amelanotic presentation was observed in two patients, both in the incidental diagnosis group. (Table 1).

Notably, in nineteen out of twenty-one cases (19/21 patients; 90.5%), patients had already undergone prior proctologic and/or dermatologic evaluations, yet the lesion had not been recognized or biopsied at that time, suggesting missed opportunities for earlier diagnosis.

Despite early suspicion, prognosis remained poor. Lymph node metastases were already present in eleven patients at diagnosis (11/21 patients; 52.4%), with similar distribution in both groups (3/5 in the early-diagnosis group A; 6/16 in the incidental group B). Distant metastases were detected in four patients (4/21 patients, 19%; 2/5 Group A and 2/16 Group B), involving the liver, lungs, and brain. (Table 1).

Initial surgical procedures varied. Wide local excision (WLE) was the most frequently performed (15/21 patients, 71.4%; 4/11 Group A; 11/16 Group B), followed by hemorrhoidectomy (3/16 Group B), and polypectomy (2/16 Group B). Only one patient underwent abdominoperineal resection (APR) - Group A, reflecting a conservative surgical approach in most cases. (Table 2).

**Table 2:** Treatment, outcome, and follow-up in Patients with Early (Group A) vs. Incidental Diagnosis of Anorectal Melanoma (Group B)

	Group A – Diagnosis suspected at first evaluation (n = 5)	Group B – Diagnosis made after surgery (n = 16)
Initial surgical procedure		
WLE	4	11
APR	1	–
Polypectomy	–	2
Hemorrhoidectomy	–	3
Resection status (Nr)		
Positive Margins	3	10
Negative Margins	2	6
Radicalization (Nr)	3	10
Adjuvant therapy, Nr	5	16
Recurrence (local or distant)	3	7
Time to recurrence, Median (IQR) (months)	4,5 (2,5)	18 (39,5)
Median follow-up (months)	13	12
Survival (Nr)	3	10
Time to mortality, Median (IQR) (months)	11 (1)	24 (17)

Comparison of clinical and prognostic features in patients with anorectal melanoma, stratified by diagnostic timing. *F* female, *M* male, *Nr* number; *WLE* Wide Local Excision; *APR* Abdominoperineal Resection.

Recurrence occurred in ten patients (10/21 patients, 47.6%; 3/5 Group A; 7/16 Group B), with a median time to recurrence of 12 months. Distant relapses were more common in patients who already had metastatic disease at the time of diagnosis, whereas local recurrences were noted primarily after WLE. (Table 2).

Median survival was 11 months in the early-diagnosis group and 24 months in the incidental group. However, due to the limited sample size, the variability in disease progression, and the incidence of advanced disease already present at diagnosis, no clear survival benefit was observed in patients diagnosed earlier. (Table 2).

## Discussion

These findings highlight the aggressive nature of anorectal melanoma (AM) and the critical need for early clinical suspicion, prompt histological diagnosis, and personalized management strategies. Although based on a small retrospective cohort, this case series highlights the complexity of diagnosis and the limitations of current treatment strategies. Our findings confirm AM as rare entity, clinically misclassified in over 76.2% of our patients (16/21 patients), often diagnosed only after surgery for presumed benign anorectal conditions [11]. Notably, in five cases the diagnosis was incidental following hemorrhoidectomy and polypectomy, and only because we routinely perform a pathologic examination after surgery [2, 12].

Even when AM was suspected at presentation, nodal metastases were found in more than 50% (3/5 patients – Group A) and distant metastases in 40% of patients (2/5 patients – Group A), confirming the disease's inherently aggressive biology. These findings suggest that while early suspicion remains essential, prognosis may not significantly improve without more effective systemic treatments. Thus, clinical awareness must be coupled with realistic expectations about outcomes.

Interestingly, in nineteen out of twenty-one cases in our series, patients had previously undergone proctologic and/or dermatologic evaluations, yet the lesion remained undiagnosed at that time. This finding underscores the silent danger of diagnostic inertia in rare diseases: AM is not necessarily absent, but simply not expected/considered. If we fail to think about it, we fail to see it. That is precisely the message of this work: consider the unexpected.

Close collaboration with dermatologists and gynecologists is essential to improve recognition of mucosal melanomas, which may be present in atypical anatomical sites such as the anal canal, perianal and genital skin. These regions should be systematically included in dermatological screening and evaluation of nevi and pigmented lesions [13]. Similar diagnostic complexity has been described for other mucosal melanomas, such as vulvar melanoma [25].

The macroscopic appearance of AM in our series, when described, included dark polypoid lesions of variable size, with a median tumor diameter of 20 mm in the Group A and 15 mm in the Group B. Most lesions were in the anal canal or anorectal junction, while only three involved the perianal skin (1/5 patients - Group A; 2/16 patients - Group B). The larger median diameter in Group A may have contributed to a more straightforward clinical recognition rather than an earlier diagnosis in itself, as more prominent or suspicious lesions are more likely to raise diagnostic concern. Conversely, the smaller size of tumors in Group B might have led to under-recognition or misdiagnosis, possibly explaining the better survival observed in this group, which could be partly due to biological factors and partly to diagnostic delay.

This finding reinforces the complementary use of anoscopy during proctological examination [26]and underscores the need for endoscopists to remain alert of this rare entity during routine assessments, particularly in cases of atypical or pigmented lesions. Routine inspection of the anal canal, even when performing flexible sigmoidoscopy, may increase the likelihood of early identification of AM, especially in patients without rectal bleeding. Notably, thrombosed internal hemorrhoids that do not resolve after 1–2 months of appropriate medical therapy should be biopsied to exclude the possibility of anorectal melanoma-cases masquerading as hemorrhoids are well described in the literature, often resulting in delayed diagnosis due to misidentification as thrombosed or prolapsed hemorrhoids. [3, 16]

From a morphological perspective, AM often appears as an intraluminal polypoid mass during rectosigmoidoscopy [27, 28]. This feature may help differentiate it from rectal adenocarcinoma, the most common anorectal malignancy [28]. Once melanoma is confirmed histologically, advanced imaging -such as contrast-enhanced CT (CECT), MRI, and PET- should be performed to evaluate tumor extent and detect systemic metastases [3, 28, 29]. Endoanal ultrasound and pelvic MRI are essential for local staging, including assessment of sphincter invasion, tumor thickness, and nodal involvement [30]. Whole-body CT and PET are critical for systemic staging, despite their limited specificity [1]. Sentinel lymph node biopsy has also been proposed as a useful tool for accurate nodal staging and postoperative treatment planning [26]. In our series, CT was used in fourteen of twenty-one patients (66.7%; 5/5 patients in the Group A; 9/16 patients in the Group B), MRI in 47.6% (3/5 patients in the Group A; 7/16 in the Group B), and PET in 33.3% (5/5 patients in the Group A; 2/16 patients in the Group B), confirming the central role of multimodal imaging in AM staging and treatment planning. The detection of mesorectal nodal metastases may not necessarily alter the initial surgical approach, as wide local excision followed by immunotherapy remains a preferred strategy when feasible. Abdominoperineal resection (Miles procedure) is currently reserved for unresectable lesions or local recurrence in the absence of distant metastases after failed immunotherapy. [31–33]

In our cohort, at the time of the diagnosis, lymph node metastases were present in 43% of patients (9/21 patients; 3/5 in Group A; 6/16 in Group B), and distant metastases were observed in 19% (4/21 patients; 2/5 in Group A; 2/16 in Group B) -regardless of whether the diagnosis had been suspected early or not. These results align with previous reports describing the high metastatic potential and poor prognosis of AM, which is often diagnosed when loco-regional or distant spread is already present [1, 5, 27].

While radical surgery such as abdominoperineal resection (APR) has traditionally been used in selected cases [30–34], our findings support a more conservative surgical approach. In 71.4% of our cases, wide local excision (WLE) was the first surgical approach. Prognosis is largely determined by the early presence of regional lymph node involvement and distant metastases, which are often present at diagnosis. This strategy appears to offer comparable oncological outcomes with respect to more radical surgery, with fewer complications and better preservation of quality of life due to the preservation of anorectal function. Moreover, compared to APR, WLE allows for an earlier use of systemic immunotherapy, which has become a cornerstone of treatment in advanced or high-risk cases, without compromising survival outcomes [31]. This point is especially relevant to consideration in the context of a disease with already limited survival prospects. In fact, prognosis is largely determined by the early presence of regional lymph node involvement and distant metastases, which are often present at diagnosis. Given the limited impact of surgical aggressiveness on survival, treatment strategies should aim to preserve function whenever practicable [31–37]. Therefore, when oncologically acceptable and technically feasible, conservative surgical approaches such as WLE are preferred, as they minimize morbidity without compromising outcomes.

Postoperative recurrence occurred in 47.6% of patients, with a median time to recurrence of 12 months. This finding raises the question of whether recurrence dynamics may be influenced by the nature of the initial diagnosis, whether it was the result of an early and precise clinical suspicion or, conversely, a delayed and incidental recognition. As previously discussed, tumor size and macroscopic features likely played a role in triggering clinical concern and shaping the timing of diagnosis, which could have downstream effects on recurrence risk and treatment planning. These results further emphasize the early onset and high rate of both local and distant relapses, highlighting the urgent need for structured care pathways and more effective targeted systemic therapies [31, 32, 36, 37].

While current systemic therapies for anorectal melanoma remain largely unsatisfactory, recent studies have highlighted encouraging results with immune checkpoint inhibitors (ICIs) and novel combinations [2, 12]. Anti-PD-1 therapies, particularly nivolumab and pembrolizumab, have demonstrated partial responses and prolonged disease control in select patients with mucosal melanomas, including AM. However, response rates remain lower than in cutaneous melanoma, likely due to the unique genetic landscape and tumor microenvironment of mucosal subtypes [1].

Combination strategies -such as anti-PD-1 with anti-CTLA-4 agents or with tyrosine kinase inhibitors (TKIs)- are currently under investigation and may offer improved efficacy. Molecular profiling may further support treatment tailoring by identifying actionable mutations (e.g., c-KIT, NRAS, or BRAF), although their prevalence is lower than in skin melanomas.

These treatments, albeit still limited by small sample sizes and lack of prospective data, underline the need to refer AM patients to high-volume centers or clinical trials, where access to innovative treatments and experimental protocols may improve outcomes [38–49].

This study has several limitations. First, its retrospective and observational design limits the ability to draw causal inferences. Second, the sample size is relatively small, reflecting the rarity of anorectal melanoma and potentially limiting the generalizability of the findings. Moreover, the limited cohort size did not allow for more complex statistical modeling, such as multivariate analysis. However, even small cohorts can offer valuable insights into diagnostic patterns and treatment outcomes for rare diseases, particularly when data are drawn from high-volume referral centers. Third, the extended inclusion period may have introduced temporal variability, but it was necessary to capture a sufficient number of cases given the exceptional rarity of this malignancy. Additionally, variability in diagnostic workup, treatment strategies, and follow-up protocols across centers may have introduced heterogeneity in the data. Finally, the lack of a centralized pathological or radiological review may have influenced staging consistency.

## Conclusion

With this work, we hope to have shed light on an often overlooked diagnosis, reminding clinicians to always consider the unexpected.

Five essential take-home messages emerge from our study:

- Anorectal melanoma (AM) should be considered in the differential diagnosis of anorectal lesions, particularly when symptoms are atypical or persistent. A meticulous proctological examination, including anoscopy, is strongly recommended. Endoscopists are also encouraged to perform routine anoscopy during lower gastrointestinal evaluations and to remain vigilant for this rare but aggressive malignancy.
- Routine skin cancer screenings should incorporate examination of mucosal and perianal areas, and dermatologists are encouraged to maintain awareness of this rare but aggressive malignancy.
- Any suspicious anorectal lesion -regardless of its apparently benign appearance- should undergo early biopsy, and all excised specimens must be submitted for histopathological analysis.
- When technically feasible, local excision should be favored over overly aggressive or mutilating procedures, given that prognosis is primarily determined by regional and distant disease spread.
- Timely referral to specialized melanoma centers and inclusion within a structured multidisciplinary care pathway (PDTA) are essential to optimize access to appropriate therapeutic strategies.

The key message is not only clinical, but cognitive: AM may remain invisible not because it is absent, but because it is not considered. “If we fail to think about it, we fail to see it.” It is rare, but the consequences of missing it are significant: diagnostic delays, advanced-stage presentation, and further limited therapeutic options. The diagnosis may be overlooked due to its rarity, its misleading presentation, often mimicking benign anorectal diseases, and because AM is already unconsidered as a possible anorectal disease by proctologists and dermatologists.
